# Case Report: Primary non-gestational small intestinal choriocarcinoma misdiagnosed as ectopic pregnancy

**DOI:** 10.3389/fonc.2026.1911597

**Published:** 2026-08-12

**Authors:** Xinjun Li, Shuo Xu, Xiangnan Zhang, Lu Sang, Yanan Ren, Ren Xu, Shihao Liu, Shengpu Wang

**Affiliations:** 1 Department of Gynecology, Hebei General Hospital, Shijiazhuang, China; 2 Department of Pathology, Hebei General Hospital, Shijiazhuang, China; 3 Department of Gynecology, The Fourth Hospital of Hebei Medical University, Shijiazhuang, China; 4 Department of Obstetrics, The Second Hospital of Hebei Medical University, Shijiazhuang, China

**Keywords:** case report, chemoresistance, ectopic pregnancy, misdiagnosis, primary small intestinal choriocarcinoma, salvage surgery

## Abstract

**Background:**

Primary small intestinal choriocarcinoma is an extremely rare nongestational malignancy associated with non-specific clinical manifestations and a high risk of misdiagnosis.

**Case presentation:**

A 40-year-old female presented with amenorrhea, vaginal bleeding, and elevated serum β-human chorionic gonadotropin (β-hCG). She was initially suspected of ectopic pregnancy and received emergency laparoscopic exploration. A 2×1 cm ileal tumor was resected, and histopathological testing verified choriocarcinoma. Given the intraoperatively normal pelvic viscera and postoperative imaging ruling out alternative primary tumors, primary small intestinal choriocarcinoma was confirmed. The patient attained complete biochemical remission after five cycles of FAV chemotherapy (fluorouracil, actinomycin D, vincristine), with serum β-hCG levels falling from 1336 mIU/mL to 0.23 mIU/mL. Thirteen months after FAV completion, biochemical relapse emerged, accompanied by pulmonary and right adnexal metastatic lesions. The adnexal lesion developed chemoresistance following nine cycles of EMA-CO (etoposide, methotrexate, actinomycin D, cyclophosphamide, vincristine) and only showed minimal response to two cycles of FAEV (fluorouracil, actinomycin D, etoposide, vincristine). Subsequent right adnexectomy was conducted, followed by two consolidation cycles of FAEV, which resulted in durable long-term remission.

**Conclusion:**

This case highlights a diagnostic pitfall in gynecological practice and suggests that multimodal therapy combining chemotherapy and surgery may achieve long-term disease control in refractory cases. Given the rarity of primary small intestinal choriocarcinoma, further molecular data are needed to validate these findings and refine therapeutic strategies.

## 1 Introduction

Choriocarcinoma is a highly malignant trophoblastic neoplasm categorized into gestational and nongestational subtypes. Nongestational choriocarcinomas are exceedingly rare, arising from germ cells or somatic cells with ectopic trophoblastic differentiation in multiple extragenital sites, including the gastrointestinal tract, lungs, and kidneys (1, 2).

Primary small intestinal choriocarcinoma is an extremely rare subtype of nongestational extragenital trophoblastic tumors (1, 2). Most reported cases present with gastrointestinal complications, including hemorrhage, obstruction, or perforation (3, 4). As this tumor secretes serum β-hCG, reproductive-aged patients may develop gynecologic symptoms such as amenorrhea and irregular vaginal bleeding, which closely mimic ectopic pregnancy or gestational trophoblastic neoplasia and may therefore result in misdiagnosis.

Furthermore, this malignancy demonstrates aggressive biological behavior with a tendency for early hematogenous dissemination. Acquired chemoresistance frequently complicates therapeutic management, contributing to a generally poor prognosis. Herein, we report a case of primary small intestinal choriocarcinoma initially misdiagnosed as ectopic pregnancy. We comprehensively detail its complete clinical course, including serial β-hCG kinetics during first-line FAV chemotherapy, biochemical relapse, subsequent distant metastasis, acquired chemoresistance, and sustained long-term remission achieved via multimodal therapy.

## 2 Case report

### 2.1 Initial presentation and misdiagnosis

A 40-year-old female (gravida 3, para 1) presented to the emergency department on March 7, 2022. Her obstetric history included cesarean delivery in 2009 for pregnancy-induced hypertension, one induced abortion, and one spontaneous miscarriage in 2014. The patient reported 51 days of amenorrhea followed by 15 days of scant, irregular vaginal bleeding and intermittent sharp hypogastric pain; no gastrointestinal symptoms were noted. Her last menstrual period occurred on January 15, 2022.

**Figure 1:**
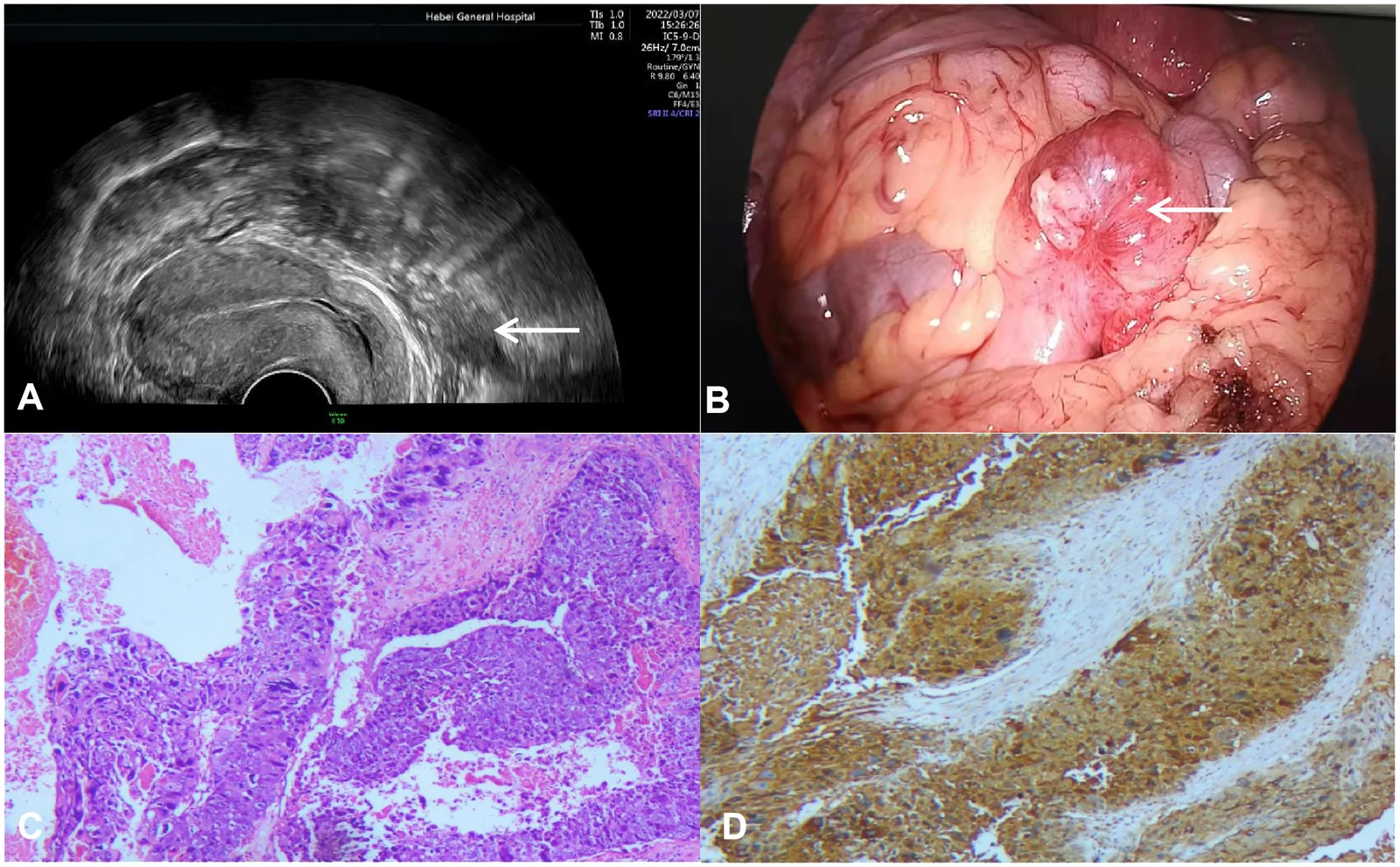
Intraoperative and pathological findings. **(A)** Transvaginal ultrasound showing a mixed-echoic mass (white arrow) adjacent to the uterine isthmus. **(B)** Intraoperative laparoscopic view showing a dark red hypervascular tumor (white arrow) on the ileal serosa. **(C)**Histopathology showing biphasic cytotrophoblasts and syncytiotrophoblasts with extensive hemorrhage (H&E staining; original magnification ×200). **(D)** Immunohistochemistry showing strong β-hCG positivity in syncytiotrophoblasts (original magnification ×200).

Admission serum β-hCG measured 2412 mIU/mL. Transvaginal ultrasonography demonstrated a thin (5 mm) homogeneous endometrium and a mixed-echoic lesion measuring 18 × 17 × 14 mm located anterolateral to the uterine isthmus (**Figure 1A**). Pelvic palpation identified a mobile, non-tender mass (3 × 2 × 2 cm) in the right anterior pelvis, anatomically separate from the uterus, without cervical motion tenderness. Based on these clinical and imaging findings, ectopic pregnancy was provisionally diagnosed, and emergency diagnostic laparoscopy was performed.

### 2.2 Surgical findings, histopathology and initial systemic treatment

Intraoperative exploration showed a macroscopically unremarkable uterus, bilateral fallopian tubes, and bilateral ovaries. A solid, dark-red nodular lesion measuring 2 × 1 cm was detected on the ileum, located approximately 75 cm proximal to the ileocecal valve (**Figure 1B**). Around 50 mL of free hemoperitoneum was observed within the rectouterine pouch. Segmental small bowel resection with primary end-to-end anastomosis was performed. Concurrent diagnostic uterine curettage retrieved no chorionic tissue or abnormal endometrial components.

Gross pathological assessment identified a 2 × 1 cm tumor nodule invading the ileal serosa. Microscopic evaluation revealed a malignant neoplasm consisting of cytotrophoblasts and syncytiotrophoblasts accompanied by prominent intratumoral hemorrhage and necrosis, with full-thickness transserosal invasion (**Figure 1C**). Immunohistochemical profiling showed diffuse positivity for pan-cytokeratin (CKpan) and β-HCG, complete absence of PAX-8 expression, and a Ki-67 proliferative index of ~70% (**Figure 1D**). These histopathological and immunohistochemical profiles established a definitive diagnosis of choriocarcinoma.

**Figure 2:**
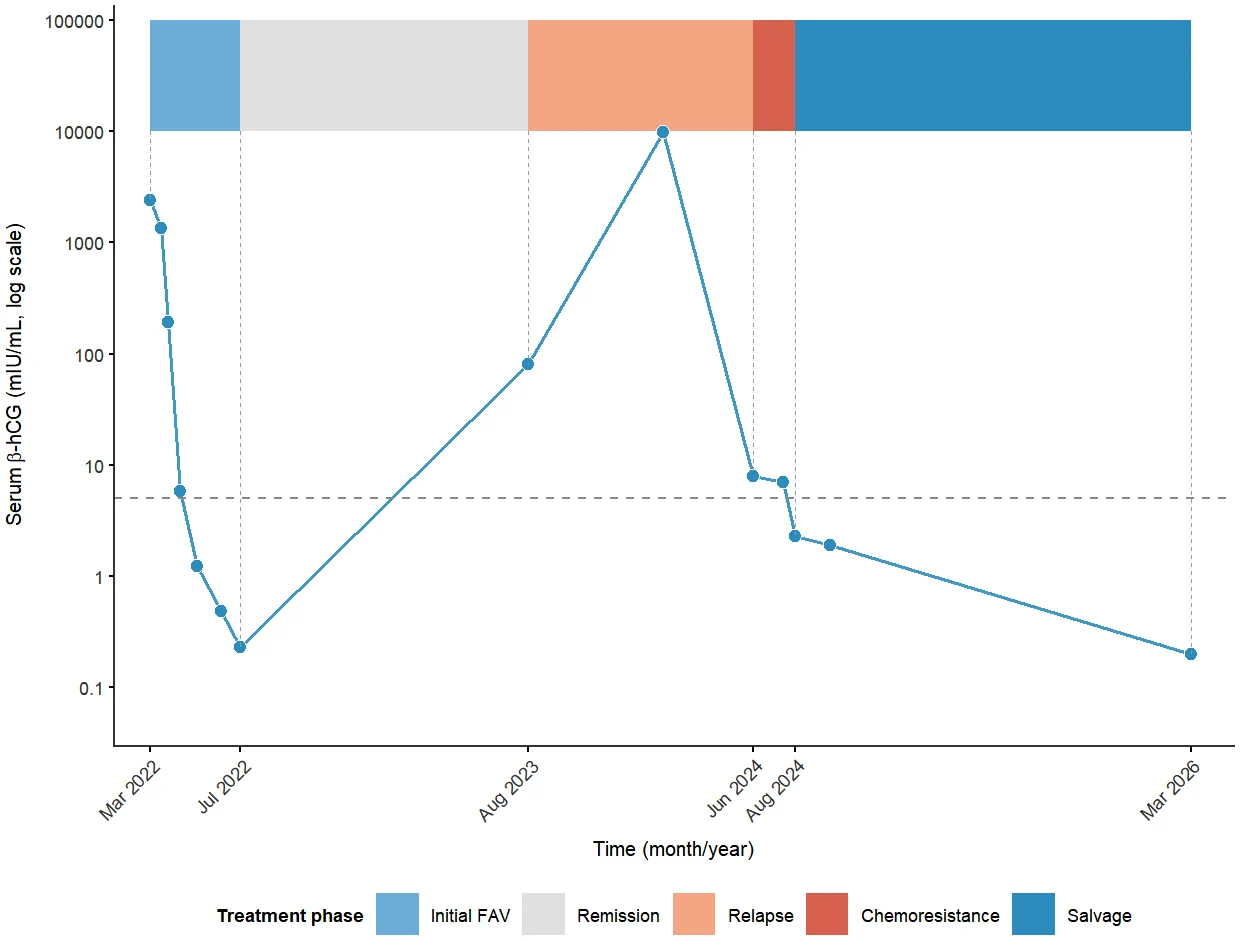
Serum β-HCG trend over the clinical course (log scale). Colored background bands denote sequential treatment phases: green (initial FAV chemotherapy), light gray (remission), orange (biochemical relapse), red (chemoresistance), and blue (salvage treatment). The horizontal dashed line at 5 mIU/mL marks the upper normal limit of β-HCG, while vertical dashed lines align measured data points with corresponding treatment stages. Key β-HCG values are annotated on the curve. All abbreviations are defined in **Table 1**.

Postoperative staging workup, including chest computed tomography and brain magnetic resonance imaging, ruled out distant metastatic lesions. According to the AJCC TNM staging system for small intestinal malignancies, the final pathological stage was determined as pT3NxM0, given the absence of lymphadenectomy and negative postoperative metastatic imaging.Serial postoperative serum β-hCG concentrations declined logarithmically throughout surveillance (**Figure 2**). On postoperative day 15 (March 22, 2022), serum β-HCG reached 1336 mIU/mL. The patient was subsequently transferred to The Second Hospital of Hebei Medical University for first-line FAV chemotherapy (fluorouracil, actinomycin D, vincristine).

Serial serum β-hCG testing confirmed a marked chemotherapeutic response: 191 mIU/mL after cycle 1 (April 1, 2022), 5.84 mIU/mL after cycle 2 (April 18, 2022), 1.22 mIU/mL after cycle 3 (May 12, 2022), 0.48 mIU/mL after cycle 4 (June 15, 2022), and 0.23 mIU/mL upon completion of five cycles (July 11, 2022), consistent with sustained complete biochemical remission.

### 2.3 Biochemical relapse, metastasis, and chemoresistance

Long-term surveillance consisted of regular serum β-hCG monitoring. Thirteen months following completion of FAV chemotherapy, biochemical relapse was detected on August 18, 2023, with serum β-hCG elevated to 81 mIU/mL and a continuously rising trend. Serial imaging assessments, including whole-body positron emission tomography-computed tomography (PET-CT, brain excluded), failed to identify radiographically apparent metastatic lesions during the early β-hCG elevation phase. By February 23, 2024, serum β-hCG had increased to 9800 mIU/mL, and repeat imaging confirmed multifocal bilateral pulmonary metastases plus a newly developed right adnexal mass. The patient was subsequently referred to Peking Union Medical College Hospital for salvage management.

Nine cycles of EMA-CO chemotherapy (etoposide, methotrexate, actinomycin D, cyclophosphamide, vincristine) were administered. Serum β-hCG decreased to 8 mIU/mL by June 28, 2024, before plateauing. Follow-up imaging demonstrated marked size reduction of pulmonary metastases, whereas the right adnexal lesion remained stable with no measurable shrinkage, indicating localized chemoresistance within the adnexal metastasis. Treatment was switched to the FAEV regimen (fluorouracil, actinomycin D, etoposide, vincristine). After two cycles (completed August 8, 2024), serum β-hCG only marginally decreased to 7 mIU/mL, confirming inadequate therapeutic response and persistent chemoresistance of the right adnexal lesion to systemic chemotherapy.

### 2.4 Salvage surgery and final remission

Following multidisciplinary consensus review (gynecologic oncology, medical oncology, radiology, and general surgery teams), right adnexectomy was performed on August 26, 2024. Postoperative histopathology verified metastatic choriocarcinoma within the resected right adnexal tissue.

Serum β-hCG rapidly normalized to <5 mIU/mL postoperatively. Two additional consolidation cycles of FAEV chemotherapy were administered to eradicate potential microscopic residual disease. Post-consolidation imaging confirmed complete resolution of all pulmonary metastatic lesions.

### 2.5 Clinical timeline

**Table 1**. Clinical timeline, treatment regimens, serum β-hCG levels, and disease status.

**Table 1:** Clinical timeline and serum β-hCG levels.

Date	Key Event	β-hCG (mIU/mL)	Status
2022-03-07	Initial resection	2412	primary diagnosis
2022-03-22	Postoperative	1336	postoperative state
2022-07-11	5 FAV cycles	0.23	complete remission (CR)
2023-08-18	Biochemical relapse	81	biochemical elevation
2024-02-23	Metastases confirmed	9800	disease progression (PD)
2024-06-28	9 EMA-CO cycles	8(plateaued)	lung metastases reduced; adnexal mass stable
2024-08-08	2 salvage FAEV cycles	7	suboptimal response to salvage therapy
2024-08-26	Right adnexectomy	<5	marker normalization post-resection
2024-10-14	2 consolidation FAEV cycles	<5	consolidation therapy completed
2026-03-02	Final follow-up	0.2	sustained complete remission (CR)

FAV, fluorouracil + actinomycin D + vincristine; EMA-CO, etoposide + methotrexate + actinomycin D + cyclophosphamide + vincristine; FAEV, fluorouracil + actinomycin D + etoposide + vincristine; mIU/mL, milli-international units per milliliter.

### 2.6 Follow-up

At the final follow-up in March 2026, serum β-hCG remained stable at 0.2 mIU/mL, and whole-body imaging showed no signs of disease recurrence. The patient maintained satisfactory overall physical status and continues scheduled long-term surveillance.

## 3 Discussion

Primary small intestinal choriocarcinoma is an extremely rare subtype of nongestational extragenital trophoblastic malignancy with atypical clinical manifestations and high misdiagnosis rates in gynecological clinical practice. In the present case, the patient presented with classic gestational-like symptoms including amenorrhea, irregular vaginal bleeding, and elevated serum β-hCG, accompanied by a pelvic soft-tissue lesion on ultrasound. These manifestations were highly consistent with the typical features of ectopic pregnancy, resulting in preoperative misdiagnosis and emergency laparoscopic exploration. This diagnostic overlap represents a critical clinical pitfall for gynecologists, as rare intestinal trophoblastic tumors are rarely included in the differential diagnosis of β-hCG-positive pelvic masses in reproductive-aged females.

### 3.1 Clinical and imaging diagnostic differentials

Given the diagnostic challenges outlined above, preoperative suspicion of primary intestinal choriocarcinoma relies heavily on imaging findings and clinical context. Cross-sectional CT or MRI can clearly delineate adhesion and invasive extension between abdominal lesions and the intestinal wall, acting as a key auxiliary modality to distinguish extragenital trophoblastic tumors. In the present case, however, preoperative CT/MRI was not ordered owing to the patient’s acute clinical presentation and provisional ultrasound diagnosis of ectopic pregnancy, which represented a critical factor leading to the preoperative diagnostic error.

Based on clinical data from this case, we propose a suggestive clinical triad to raise suspicion of primary intestinal nongestational choriocarcinoma in β-hCG-positive reproductive-aged women: (1) elevated serum β-hCG without a verifiable recent gestational event; (2) intact, tumor-free uterus and bilateral adnexa confirmed by intraoperative exploration or imaging; (3) an unexplained intestinal mass accompanied by amenorrhea or irregular vaginal bleeding typical of gestational disease. This composite profile helps differentiate intestinal choriocarcinoma from ectopic pregnancy and primary pelvic trophoblastic malignancies.

Notably, the absence of chorionic villi on diagnostic uterine curettage, a routine marker to rule out intrauterine pregnancy, lacks sufficient specificity to confirm an intestinal primary site. This pathological feature can also be observed in primary ovarian or fallopian tube choriocarcinoma without intrauterine trophoblastic implantation. Accordingly, curettage histology alone cannot accurately localize the tumor origin and should only be regarded as supplementary rather than definitive diagnostic evidence (5).

### 3.2 Pathogenic hypotheses

The pathogenesis of extragonadal nongestational choriocarcinoma, including lesions arising within the small intestine, remains incompletely elucidated. Two prevailing pathogenic hypotheses have been proposed to explain the development of this rare neoplasm (6). The first mechanism involves aberrant ectopic migration of primordial germ cells: during embryonic development, primordial germ cells diverge from their canonical migratory route, implant ectopically within the intestinal mesentery or bowel wall, and remain dormant for decades until somatic mutations or alterations in the local tissue microenvironment drive malignant transformation in adulthood (7). The second theory centers on somatic transdifferentiation, whereby preexisting intestinal epithelial malignancies (e.g., intestinal adenocarcinoma) acquire trophoblastic differentiation capacity and undergo dedifferentiation to form pure choriocarcinoma components (7).

In the present case, the lesion manifested as a solitary ileal mass without histopathologically identifiable adenomatous or carcinomatous precursor tissue, a morphological feature consistent with the germ cell ectopia hypothesis. Nevertheless, owing to the extraordinary rarity of this tumor subtype and the lack of confirmatory molecular genetic profiling in our specimen, all mechanistic inferences drawn herein remain purely speculative and cannot be extrapolated to broader patient populations.

### 3.3 Response to first-line FAV chemotherapy

FAV is a fluorouracil-based regimen widely used for low-to-medium risk gestational trophoblastic disease in China (10). In the present case, five cycles of first-line FAV induced a rapid, stepwise decline in serum β-hCG to complete biochemical remission (0.23 mIU/mL), with serial measurements of 191, 5.84, 1.22, 0.48, and 0.23 mIU/mL after cycles 1–5, providing detailed longitudinal response data for this regimen in primary intestinal choriocarcinoma. Nevertheless, this observation derives from a single case and should not be overgeneralized.

### 3.4 Biochemical relapse, metastatic progression, and site-specific chemoresistance

Thirteen months after complete remission, biochemical relapse occurred with isolated serum β-hCG elevation (81 mIU/mL) and negative whole-body PET-CT, indicating serological relapse precedes radiological detectability. When β-hCG rose to 9800 mIU/mL, imaging confirmed progressive pulmonary and adnexal metastases.

Nine cycles of EMA-CO induced marked regression of pulmonary lesions, but the adnexal mass persisted with β-hCG plateauing at 8 mIU/mL, indicating site-specific acquired chemoresistance (8). This pattern differs from gestational choriocarcinoma, which typically shows uniform chemosensitivity with rare focal resistance (4). FAEV salvage chemotherapy only marginally reduced β-hCG to 7 mIU/mL, confirming lesion refractoriness.

Importantly, divergent chemosensitivity between metastatic sites does not confirm a nongestational origin, as intratumoral heterogeneity may also account for this finding (9). Thus, an intestinal primary lesion with focal resistance remains suggestive of, but not definitive for, a nongestational origin.

### 3.5 Therapeutic role of salvage resection for resistant metastatic disease

When systemic EMA-CO effectively controlled pulmonary metastases but failed to eradicate the chemoresistant adnexal lesion, and subsequent FAEV produced only marginal benefit, surgical resection of the isolated resistant metastasis was performed. Postoperatively, serum β-hCG normalized rapidly to <5 mIU/mL, confirming successful elimination of the primary drug-resistant tumor source. This case suggests that for trophoblastic tumors with focal chemoresistance, salvage surgery combined with systemic chemotherapy may reverse therapeutic failure and may enable sustained disease control (9).

### 3.6 Clinical rationale for FAV and FAEV regimens

FAV and FAEV are fluorouracil-based regimens developed in China for trophoblastic neoplasia. FAEV has shown a 73.6% initial complete remission rate for high-risk gestational trophoblastic disease (10, 11). In this case, FAEV showed limited efficacy against refractory disease but provided potent consolidation activity after surgical resection of the resistant lesion, contributing to durable remission (β-hCG 0.2 mIU/mL at last follow-up). This observation suggests FAEV may be a useful post-surgical consolidation option in selected patients with focal chemoresistance, though its role in nongestational choriocarcinoma warrants further study (8).

### 3.7 Key clinical insights

This case provides the following preliminary clinical insights:(1)Prolonged, rigorous β-hCG surveillance after complete remission is warranted, as biochemical relapse may precede radiologic detectable metastasis by months.(2)Prompt switch to second-line regimens (EMA-CO, FAEV) should be considered upon first-line treatment failure (5, 10).(3)Discordant response across metastatic sites suggests possible site-specific chemoresistance (4, 12).(4)Surgical resection is a valuable salvage option for isolated, chemoresistant metastatic lesions (12).(5)FAV and FAEV regimens developed in China show favorable efficacy for both first-line induction and post-surgical consolidation (10, 11, 13).

### 3.8 Study limitations

The primary limitation is the absence of short tandem repeat (STR) genotyping—the gold standard for distinguishing nongestational from gestational choriocarcinoma (2). Archived primary tumor specimens were unavailable for retrospective testing, precluding definitive subtype classification. While clinical features (no recent gestation, normal pelvic organs on intraoperative exploration, negative curettage for villi, site-specific chemoresistance) support a nongestational origin, they cannot fully exclude occult gestational choriocarcinoma with intestinal metastasis, or early spread from an occult microscopic ovarian primary that regrew after treatment cessation.

Nevertheless, two independent clinical lines of evidence render the occult ovarian primary hypothesis less plausible. First, intraoperative laparoscopic exploration, as well as imaging studies performed preoperatively, postoperatively, and at biochemical relapse, all revealed no macroscopic ovarian lesions. Second, the pulmonary metastases responded markedly to EMA-CO chemotherapy while the adnexal lesion remained refractory; if both metastatic lesions arose from a single ovarian primary tumor, their chemosensitivity patterns would theoretically be more consistent. The absence of macroscopic ovarian lesions and this divergent chemosensitivity across metastatic sites still favor a primary small intestinal origin. Upfront STR genotyping is recommended for future cases to confirm tumor origin (2, 5).

## 4 Conclusion

Primary small intestinal choriocarcinoma easily mimics ectopic pregnancy in reproductive-age females. This case illustrates that its clinical course can be complicated by delayed biochemical relapse, metastatic spread, and the development of site-specific chemoresistance. Multimodal management integrating systemic chemotherapy and surgical resection of isolated resistant lesions may yield durable disease control for relapsed, chemoresistant cases. Definitive diagnosis depends on comprehensive clinicopathological findings without molecular testing; whenever feasible, upfront STR genotyping is recommended to verify the tumor’s origin (5, 12).

## 5 Patient’s perspective

The patient thanked the clinical team for prompt diagnosis and multidisciplinary treatment. The period of biochemical relapse represented her greatest psychological burden: serial serum β-HCG elevated persistently, while cross-sectional imaging revealed no detectable metastatic foci. Her quote highlighted this distress: “Not knowing where the cancer was hiding was more stressful than the treatment itself.” Major chemotherapy toxicities including fatigue and myelosuppression were effectively alleviated via supportive interventions. She has fully recovered daily function and maintains consistent long-term surveillance. With written informed consent, she consented to case publication, hoping her clinical journey may benefit patients affected by identical rare trophoblastic neoplasms.

## Data Availability

The original contributions presented in the study are included in the article/supplementary material. Further inquiries can be directed to the corresponding author.
